# Treatment and outcomes for patients with relapsed or refractory diffuse large B-cell lymphoma: a contemporary, nationwide, population-based study in the Netherlands

**DOI:** 10.1038/s41408-023-00970-z

**Published:** 2024-01-04

**Authors:** Elise R. A. Pennings, Müjde Durmaz, Otto Visser, Eduardus F. M. Posthuma, Djamila E. Issa, Martine E. D. Chamuleau, Pieternella J. Lugtenburg, Marie José Kersten, Avinash G. Dinmohamed

**Affiliations:** 1 Department of Hematology, Amsterdam UMC location University of Amsterdam, Amsterdam, The Netherlands; 2 Cancer Center Amsterdam, Amsterdam, The Netherlands; 3 LYMMCARE (Lymphoma and Myeloma Center Amsterdam), Amsterdam, The Netherlands; 4 Erasmus School of Health Policy and Management, Erasmus University Rotterdam, Rotterdam, The Netherlands; 5 Department of Research and Development, Netherlands Comprehensive Cancer Organisation (IKNL), Utrecht, The Netherlands; 6 Department of Registration, Netherlands Comprehensive Cancer Organisation (IKNL), Utrecht, The Netherlands; 7 Department of Hematology, Leiden University Medical Center, Leiden, The Netherlands; 8 Department of Internal Medicine, Reinier de Graaf Gasthuis, Delft, The Netherlands; 9 Department of Internal Medicine, Jeroen Bosch Hospital, Den Bosch, The Netherlands; 10 Department of Hematology, Amsterdam UMC location Vrije Universiteit Amsterdam, Amsterdam, The Netherlands; 11 Department of Hematology, Erasmus MC Cancer Institute, University Medical Center Rotterdam, Rotterdam, The Netherlands; 12 Department of Public Health, Erasmus MC, University Medical Center Rotterdam, Rotterdam, The Netherlands

**Keywords:** B-cell lymphoma, Epidemiology

Diffuse large B-cell lymphoma (DLBCL) is a common and aggressive form of non-Hodgkin lymphoma primarily affecting adults. The introduction of rituximab―a monoclonal antibody that targets the CD20 antigen on B-cells―in the early 2000s revolutionized DLBCL treatment, substantially enhancing survival outcomes [1–3]. Currently, rituximab combined with cyclophosphamide, doxorubicin, vincristine, and prednisone (R-CHOP) constitutes the standard first-line treatment for DLBCL [4]. However, 20–40% of patients experience relapsed or refractory (R/R) disease following initial R-CHOP treatment, and subsequent chemo(immuno)therapy regimens―with or without stem cell transplantation―have limited efficacy [3–7]. Consequently, most R/R DLBCL patients face a dismal prognosis, although recent advances, including chimeric antigen receptor (CAR) T-cell therapy and bispecific antibodies, demonstrate promising efficacy for select patient subsets [4, 7].

The SCHOLAR-1 study established a benchmark for recent single-arm phase II clinical trials in R/R DLBCL reporting outcomes for 636 patients with refractory DLBCL, including transformed follicular lymphoma (tFL) and primary mediastinal B cell lymphoma (PMBCL), based on data from two randomized trials and two academic databases [7]. In SCHOLAR-1 refractory DLBCL was defined as stable disease (SD) or progressive disease (PD) as the best response to first- or later-line therapy or relapse ≤12 months after autologous stem cell transplantation (autoSCT). This study identified an objective response rate of 26%, with a mere 7% achieving complete remission (CR) to treatment for R/R disease. Furthermore, the median overall survival (OS) was 6.3 months, and the 2-year OS rate was only 20% [7]. Nevertheless, given its non-population-based design, extrapolating SCHOLAR-1 results to a real-world population demands caution. Since, population-based studies confirming findings from SCHOLAR-1 are scarce, we aimed to validate SCHOLAR-1 outcomes in patients with DLBCL (including tFL and PMBCL) within contemporary clinical practice in the Netherlands in a nationwide population-based study [8].

We selected all adult ( ≥18 years) patients diagnosed with DLBCL, tFL, and PMBCL between January 1, 2014, and December 31, 2018―using the International Classification of Diseases for Oncology morphology codes 9679 and 9680―from the Netherlands Cancer Registry (NCR) [9]. The NCR was established in 1989 and includes all newly diagnosed malignancies in the Netherlands [10]. Since 2014, the NCR collects more detailed data on hematological malignancies, including specific disease characteristics and the exact first-line therapeutic regimens. Case notifications are received through the Nationwide Network of Histo and Cytopathology and the Nationwide Registry of Hospital Discharges. After case notification, trained NCR data managers collect data on diagnosis and first-line treatment through retrospective medical records review. For this study we additionally gathered follow-up on subsequent treatment and outcomes with ≥3 years of follow-up post-diagnosis. According to the Central Committee on Research involving Human Subjects (CCMO), this type of observational study does not require approval from an ethics committee in the Netherlands. The Privacy Review Board of the NCR approved using anonymous data for this study. Inclusion criteria similar to SCHOLAR-1 were used: (i) diagnosis of DLBCL, tFL, and PMBCL, (ii) first-line treatment with an anti-CD20 monoclonal antibody and an anthracycline, (iii) refractory disease―defined as SD (SD as the best response to ≥3 cycles of first-line treatment or ≥2 cycles of second- or later-line treatment) or PD as the best response to therapy― or relapse ≤12 months after autoSCT, and (iv) initiation of subsequent treatment―defined as subsequent chemo- and/or immunotherapy for treatment of DLBCL, tFL or PMBCL― at the first instance of R/R disease. To facilitate comparison with SCHOLAR-1, patients receiving CAR T-cell therapy in any treatment line were excluded. Patients were categorized into three subgroups based on the first instance of R/R disease: primary refractory, refractory to ≥second-line treatment, and relapsed ≤12 months post-autoSCT.

Descriptive statistics and the Kaplan-Meier method were used to analyze patient characteristics and survival, respectively, following the initiation of treatment for the first instance of R/R disease. OS was measured until death or the end of follow-up, while event-free survival (EFS) was measured until progression, subsequent treatment initiation, death, or the end of follow-up, whichever occurred first. Survival differences across R/R disease subgroups were evaluated using the log-rank test, with *P-*values <0.05 considered significant. Cox proportional hazards regression models were used to assess the association of age, sex, diagnosis, Ann Arbor stage, International Prognostic Index (IPI) score and R/R disease subgroup with OS and EFS. Variables with a *P*-value <0.1 in univariable analyses were selected for the multivariable models. Statistical analyses were conducted using STATA Statistical Software version 17.0 (StataCorp, College Station, TX).

Between 2014 and 2018, 6899 adult patients were diagnosed with DLBCL, tFL, and PMBCL in the Netherlands. Of these, 455 (7%) met the aforementioned (i–iii) inclusion criteria for R/R disease (Supplementary Fig. 1). Notably, around 50% of these 455 patients did not receive subsequent treatment at the first instance of R/R disease, yielding 225 (49%) patients meeting all inclusion criteria (i–iv) (Supplementary Fig. 1). Treatment patterns and sequencing across different lines of therapy are visualized in a Sankey plot (Fig. 1A). Nearly all patients received R-CHOP as first-line treatment, with 71% continuing to intensive second-line regimens, of whom only 29% underwent autoSCT.

**Fig. 1:**
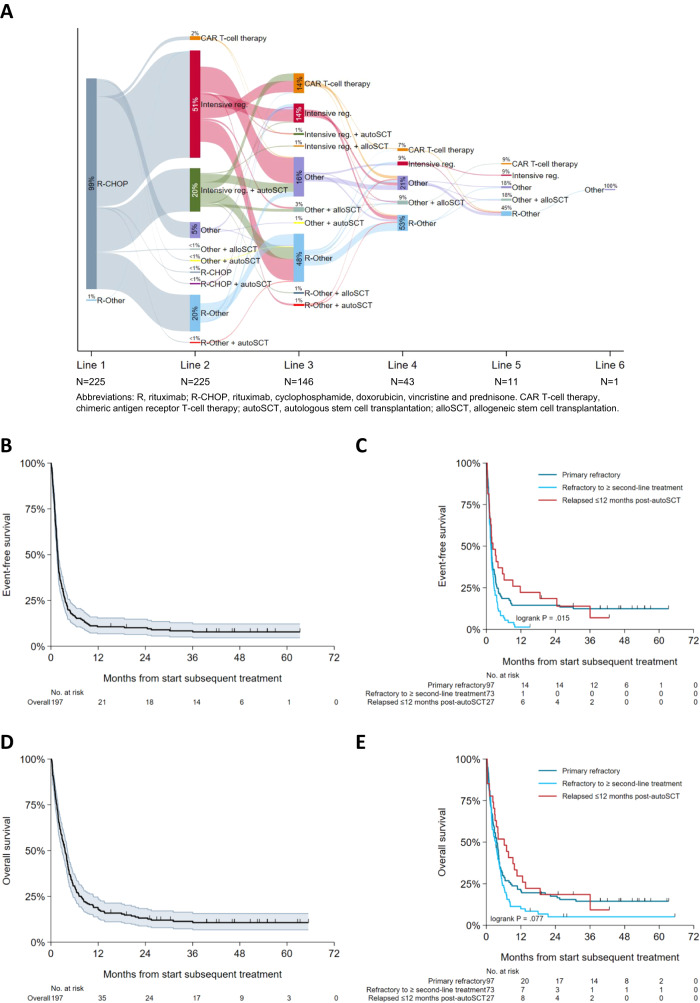
Treatment and survival of patients with relapsed or refractory (R/R) diffuse large B-cell lymphoma (DLBCL). Treatment across lines of therapy for patients with R/R DLBCL (**A**) and event-free survival (EFS) and overall survival (OS) from start of subsequent treatment shown for the total cohort (**B**, **D**, respectively) and per R/R disease subgroup (**C**, **E**, respectively). The intensive regimens group consists of high-dose salvage chemotherapy regimens such as R-DHAP, R-VIM, R-DHAP/VIM, R-IGEV, R-GDP, or R-ICE and normally patients with a response continue with auto-SCT. The R-other group contains regimens such as R-PECC or R-bendamustine. The other group contains regimens such as lenalidomide, brentuximab vedotin, pixantrone, selinexor or PECC without rituximab. *R-DHAP* rituximab, dexamethasone, high-dose cytarabine (Ara-C), cisplatin, *R-VIM* rituximab, etoposide, ifosfamide, methotrexate, *R-IGEV* rituximab, ifosfamide, gemcitabine, vinorelbine, *R-GDP* rituximab, gemcitabine, cisplatin, dexamethasone, *R-ICE* rituximab, ifosfamide, carboplatin, etoposide, *(R-)PECC* (rituximab), prednisolone, etoposide, chlorambucil, lomustine, *R-bendamustine* rituximab, bendamustine.

After excluding 28 patients who received CAR T-cell therapy, our analytical cohort comprised 197 patients (median age, 62 years; 63% male; 69% Ann Arbor stage III–IV), primarily with DLBCL (87%), followed by tFL (10%) and PMBCL (4%). Patient characteristics are shown in Table 1. Forty-nine percent had primary refractory disease, 37% was refractory to ≥second-line treatment and 14% relapsed ≤12 months post-autoSCT.

**Table 1:** Patient characteristics of our study compared to SCHOLAR-1.

Characteristics	NCR cohort		SCHOLAR-1^7^	
	N	(%)	N	(%)
Total no. of patients	197	(100)	636	(100)
Sex, male	125	(63)		64
Age, years				
Median (min-max)	62 (18–81)		55 (19–81)	
18–60	87	(44)		NA
61–69	64	(32)		NA
≥70	46	(23)		NA
Primary diagnosis				
DLBCL	171	(87)		87
tFL	19	(10)		4
PMBCL	7	(4)		2
Indeterminate/ missing	0	(0)		7
Disease stage				
I–II	36	(18)		27
III–IV	135	(69)		72
Unknown	26	(13)		<1
ECOG performance status				
0–1	55	(28)		73
2–4	21	(11)		14
Unknown	121	(61)		13
IPI risk classification^**a**^				
Low	48	(24)		25
Low-intermediate	54	(27)		24
High-intermediate to high	95	(48)		33
Missing or incompletely assessed^b^	119	(60)		18
R/R disease category				
Primary refractory	97	(49)		28
Refractory to ≥second-line treatment	73	(37)		50
Relapsed ≤12 months post-autoSCT	27	(14)		22
Prior systemic therapy lines^**c**^				
1	97	(49)		28
2–3	73	(37)		49
≥4	0	(0)		<1
Center of treatment				
Non-academic	131	(66)		NA
Academic	66	(34)		NA

*DLBCL* diffuse large B-cell lymphoma, *tFL* transformed follicular lymphoma, *PMBCL* primary mediastinal B-cell lymphoma, *IPI* International Prognostic Index, *R/R* relapsed or refractory, autoSCT autologous stem cell transplantation

^a^In the advent of a missing IPI risk parameter, that parameter was regarded as negative (i.e. no point assigned) in the NCR dataset. Therefore, an IPI score could be calculated for all patients.

^b^Percentage of patients in whom one or more IPI risk parameters were missing or unknown.

^c^Similar to SCHOLAR-1, information on prior systemic therapy lines is only presented for the 86% (*n* = 170) of patients who were refractory to systemic therapy and not for the patients who relapsed post-autoSCT (*n* = 27). Percentages in the Table are shown as percentage of the total cohort (*N* = 197). Of the 170 patients who were refractory to systemic treatment, 57% (*n* = 97) received 1 prior systemic therapy line and 43% (*n* = 73) received 2-3 prior systemic therapy lines.

The best response to subsequent treatment at the first instance of R/R disease was assessed for 140 patients (71%), with overall response rates (ORR) and CR rates (CRR) of 21% (*N* = 41; 95% confidence interval [CI], 15-27%) and 9% (*N* = 18; 95% CI, 6-14%), respectively. The highest ORR and CRR were observed in patients relapsing within 12 months post-autoSCT: 26% and 15%, respectively, but only the differences in CRR between the subgroups were statistically significant (ORR: *P* = 0.282, CRR: *P* = 0.005; Supplementary Fig. 2).

At a median follow-up of 3.5 months (interquartile range [IQR]: 1.5–7.1; median follow-up of patients alive: 45.9 months (IQR: 27.8-54.8)), the median EFS from subsequent treatment initiation was 1.7 months (95% CI, 1.5–1.9), with a 2-year EFS rate of 10% (95% CI, 6–15%; Fig. 1B). The median EFS for the three R/R disease subgroups was 1.8 months (95% CI, 1.4–2.0), 1.6 (95% CI, 1.3–1.9), and 2.3 (95% CI, 1.1–6.2), months, with respective 2-year EFS rates of 14% (95% CI, 8–22%), 0% and 19% (95% CI, 7–35%) (*P* = 0.015; Fig. 1C). The median OS from subsequent treatment initiation was 3.6 months (95% CI, 2.8–4.2), with a 2-year OS rate of 13% (95% CI, 9-18%; Fig. 1D). OS across the three R/R disease subgroups did not significantly differ (*P* = 0.077), with a median OS of 3.6 (95% CI, 2.5-4.3), 3.3 (95% CI, 1.9-4.3), and 6.2 months (95% CI, 2.5–10.6), respectively. The corresponding 2-year OS rates were 18% (95% CI, 11–26%), 5% (95% CI, 1–12%), and 19% (95% CI, 7–35%), respectively (Fig. 1E). Patient characteristics of the 17 patients with long-term survival (alive ≥36 months from subsequent treatment initiation; Fig. 1D) are provided in Supplementary Table 1. Response to subsequent treatment was assessed for 16/17 patients, with an ORR and CRR of 65% (*n* = 11) and 53% (*n* = 9), respectively.

In univariable analyses, a higher Ann Arbor stage (stage III–IV) and IPI score, and being refractory to ≥second-line treatment were significantly associated with both worse OS and EFS, and male sex solely with worse EFS. In multivariable analyses only a high-intermediate to high IPI score was significantly associated with worse OS (Hazard ratio: 2.24 (95% CI, 1.20–4.18)) and EFS (Hazard ratio: 1.98 (95% CI, 1.08–3.61)) (Supplementary Table 2 & Supplementary Fig. 3).

This study is among the first population-based studies validating SCHOLAR-1 results in adult R/R DLBCL patients treated in the modern rituximab era, offering a real-world perspective and elucidating marked disparities between patients treated in clinical trials or academic centers and routine clinical practice. Remarkably, 230 (51%) of the 455 patients who met R/R disease criteria in our study had to be excluded as they did not receive subsequent treatment at the first instance of R/R disease (Supplementary Fig. 1). Notably, these patients had worse OS measured from the first instance of R/R disease (median OS: 1.6 months) than those included in the study (median OS: 5.0 months) (Supplementary Table 3 & Supplementary Fig. 4).

Compared to SCHOLAR-1, our population-based study found a lower best ORR (21% (95% CI, 15–27%) vs. 26% (95% CI, 21–31%)), and a slightly higher CRR (9% (95% CI, 6–14%) vs. 7% (95% CI, 3–15%)). Also, the median OS was lower; 3.6 months (95% CI, 2.8-4.2) vs. 6.3 months (95% CI, 5.9–7.0), as was the 2-year OS rate; 13% (95% CI, 9–18%) vs. 20% (95% CI, 16–23%). Additionally, our cohort showed worse OS for patients with refractory disease than SCHOLAR-1. Patients with primary refractory disease had a median OS of 3.6 (95% CI, 2.5–4.3) vs. 7.1 (95% CI, 6.0–8.1) months and a corresponding 2-year OS rate of 18% (95% CI, 11-26%) vs. 24% (95% CI, 18–30%), and patients refractory to ≥second-line treatment had a median OS of 3.3 (95% CI, 1.9–4.3) vs. 6.1 (95% CI, 5.2–7.0) months, with a respective 2-year OS rate of 5% (95% CI, 1–12%) vs. 17% (95% CI, 13–22%). On the other hand, OS for patients relapsing ≤12 months after autoSCT was similar to SCHOLAR-1 (median OS: 6.2 months (95% CI, 2.5–10.6 vs 95% CI, 5.2–7.6); 2-year OS rate: 19% (95% CI, 7–35% vs 95% CI, 12–27%)) [7]. As the CIs for the response rates and most 2-year OS rates overlap, it should be noted that these differences in outcomes between our study and SCHOLAR-1 might not be statistically significantly different. The higher median age, IPI score, and incidence of primary refractory disease in our cohort compared to SCHOLAR-1 could possibly explain the significant differences in outcomes, although only a higher IPI score was significantly associated with worse survival in our cohort. Importantly, the majority of patients (66%) in our study were treated in non-academic centers (patient characteristics according to center of treatment are provided in Supplementary Table 4). This finding underscores the importance of validating clinical trial or academic database results using population-based data to contextualize patient outcomes in daily practice.

In addition to validating SCHOLAR-1 results, we estimated EFS for our cohort, a crucial measure in patient counseling and clinical trial endpoints [6]. We found a median EFS of 1.7 months and a 2-year EFS rate of 10%, with significant differences across R/R disease subgroups (patient characteristics per subgroup are provided in Supplementary Table 5).

A previous population-based study by Daneels and colleagues in 2022 offered some information on first- and second-line treatments and OS estimates in R/R DLBCL using Belgian Cancer Registry data [8]. However, that study (i) could not fully align with SCHOLAR-1 inclusion criteria, (ii) did not provide information on response status, and (iii) could not derive the exact timing of R/R disease, thereby limiting comparison and potentially incurring selection bias [8]. Furthermore, Harrysson et al., and Arboe et al. presented population-based outcomes of R/R DLBCL patients in Sweden and Denmark, respectively [11, 12]. Besides patients who were primary refractory, these studies also included patients who initially responded to first-line treatment but subsequently relapsed. In our cohort patients who relapsed after initially responding to first-line treatment were only included if they were refractory to ≥second-line treatment or relapsed ≤12 months post-autoSCT. In addition, both studies measured survival from date of R/R disease instead of start of subsequent treatment. These differences in the R/R disease definition and survival measurement hamper the comparability of outcomes between these two studies and our study. Only Harrysson et al. reported separate survival outcomes for patients with primary refractory disease and found a median OS of 4.4 months (95% CI, 3.8–5.0) and a 2-year OS rate of 14% (95% CI, 10–19%). However, they also included patients who did not receive subsequent treatment possibly explaining the worse 2-year OS rate compared to our cohort [11].

Our study’s main strength is using nationwide population-based data, providing non-selected outcomes for R/R DLBCL, including tFL and PMBCL, with detailed information on later-line therapeutic regimens. These insights in daily clinical practice can guide clinical decisions and research directions. For example, this study shows that only 29% of the patients with R/R DLBCL who received intensive second-line regimens underwent autoSCT, highlighting the need for optimized treatment strategies. Furthermore, EFS data is available, an essential measure reflecting the effect of subsequent treatment on survival.

Limitations include an incomplete IPI-score, mainly due to the lack of Eastern Cooperative Oncology Group (ECOG) performance scores for many patients. This also hampered to adequately assess the association of ECOG performance scores with survival. Additionally, unlike SCHOLAR-1, our study, as per Dutch clinical practice, defined SD as the best response to ≥ three cycles of first-line therapy rather than ≥ four cycles as was used in SCHOLAR-1 [7]. Lastly, a small subset of patients receiving treatment in a clinical trial is also included in this analysis, although this is inherent in a population-based setting (i.e., real world).

Notwithstanding these limitations, our results emphasize the need to improve the dismal prognosis of patients with R/R DLBCL, including tFL and PMBCL. Encouragingly, recent advances, such as CAR T-cell therapy and bispecific antibodies, have the potential to improve outcomes and reshape the treatment landscape of R/R DLBCL [4]. Since May 2020, CAR T-cell therapy is reimbursed and standard of care for adult patients with R/R DLBCL after ≥2 lines of systemic therapy in the Netherlands, which significantly improved survival outcomes for these patients [13]. Nowadays, at least 79% (*n* = 156/197) of the included patients in our cohort meet this indication and would have been potentially eligible for treatment with CAR T-cell therapy as standard of care. However, a recently published population-based study showed that only a selected subset will eventually receive CAR T-cell therapy, with rapidly progressive disease being the main reason for ineligibility [13]. The real-world data in this study support future research (e.g., benchmarking, calculating population estimates) and facilitate comparison of (cost-)effectiveness of novel treatments with usual care for patients with R/R DLBCL in clinical practice [3, 14].

### Supplementary information

**Supplementary appendix**

## Data Availability

The data that support the findings of this study are available via The Netherlands Comprehensive Cancer Organisation. These data are not publicly available, and restrictions apply to the availability of the data used for the current study. However, these data are available upon reasonable request and with permission of The Netherlands Comprehensive Cancer Organisation.
