# Supplementary material for: Treatment and outcomes for patients with relapsed or refractory diffuse large B-cell lymphoma: a contemporary, nationwide, population-based study in the Netherlands
Source: Blood Cancer J. 2024 Jan 4;14(1):3. doi: 10.1038/s41408-023-00970-z (PMC10766619; doi:10.1038/s41408-023-00970-z)
Supplement: Supplementary file 1 — Supplementary appendix [file 41408_2023_970_MOESM1_ESM.pdf]

SUPPLEMENTARY APPENDIX

Title

Treatment and outcomes for patients with relapsed or refractory diffuse large B-cell lymphoma: a contemporary, nationwide, population-based study in the Netherlands

Authors and affiliation

Elise R.A. Pennings[1,2,3,4]* & Müjde Durmaz[1,5]*, Otto Visser[6], Eduardus F.M. Posthuma[7,8], Djamila E. Issa[9], Martine E.D. Chamuleau[2,10], Pieternella J. Lugtenburg[11], Marie José Kersten[1,2,3], and Avinash G. Dinmohamed[1,2,3,5,10,12]

[1]Department of Hematology, Amsterdam UMC location University of Amsterdam, Amsterdam, The Netherlands; [2]Cancer Center Amsterdam, Amsterdam, The Netherlands; [3]LYMMCARE (Lymphoma and Myeloma Center Amsterdam), Amsterdam, The Netherlands; [4]Erasmus School of Health Policy and Management, Erasmus University Rotterdam, Rotterdam, The Netherlands; [5]Department of Research and Development, Netherlands Comprehensive Cancer Organisation (IKNL), Utrecht, The Netherlands; [6]Department of Registration, Netherlands Comprehensive Cancer Organisation (IKNL), Utrecht, The Netherlands; [7]Department of Hematology, Leiden University Medical Center, Leiden, The Netherlands; [8]Department of Internal Medicine, Reinier de Graaf Gasthuis, Delft, The Netherlands; [9]Department of Internal Medicine, Jeroen Bosch Hospital, Den Bosch, The Netherlands; [10]Department of Hematology, Amsterdam UMC location Vrije Universiteit Amsterdam, Amsterdam, The Netherlands; [11]Department of Hematology, Erasmus MC Cancer Institute, University Medical Center Rotterdam, Rotterdam, The Netherlands; [12]Department of Public Health, Erasmus MC, University Medical Center Rotterdam, Rotterdam, The Netherlands

*These authors contributed equally to this work

Supplemental Figure 1

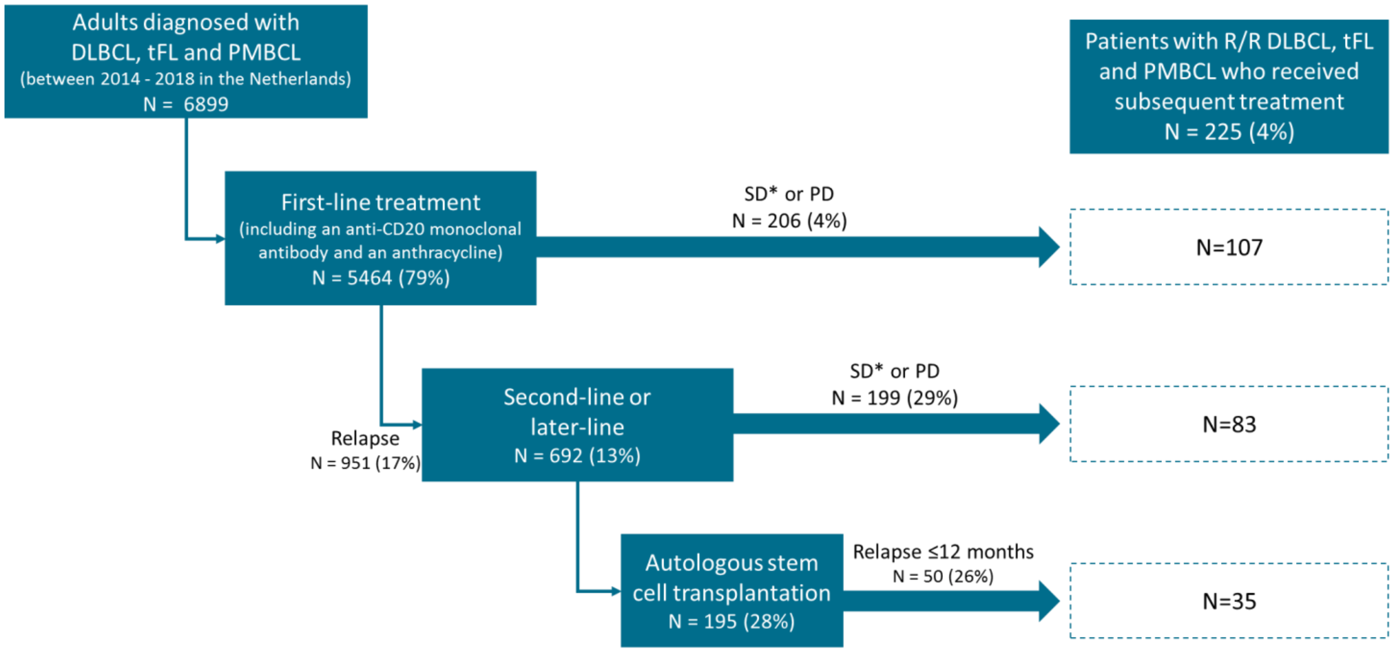

*SD as best response to ≥ 3 cycles of first-line treatment or ≥ 2 cycles of second- or later-line treatment

Abbreviations: DLBCL, diffuse large B-cell lymphoma; tFL, transformed follicular lymphoma; PMBCL, primary mediastinal B cell lymphoma; SD, stable disease; PD, progressive disease; R/R, relapsed or refractory.

Supplemental Figure 2

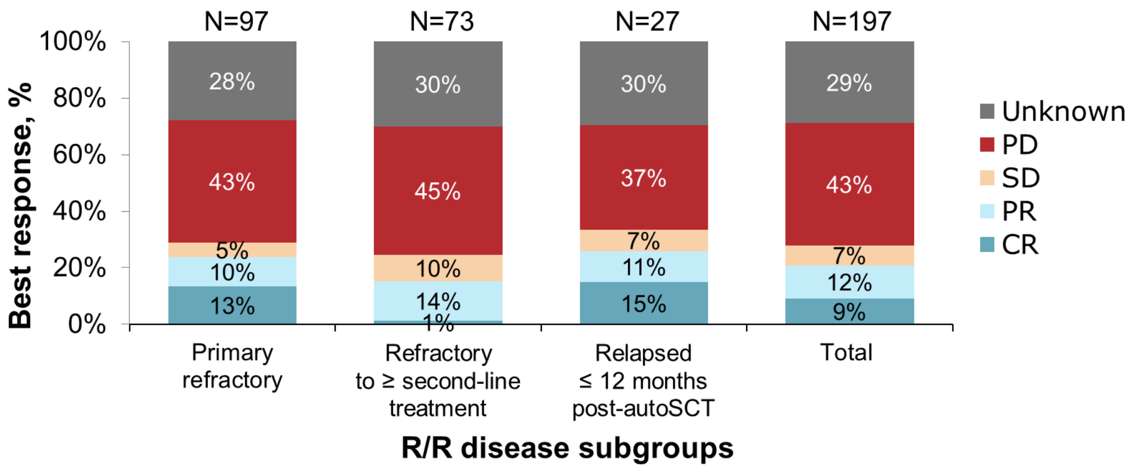

*Due to rounding, percentages may not always count up to 100%.
Abbreviations: R/R, relapsed or refractory; autoSCT, autologous stem cell transplantation; PD, progressive disease; SD, stable disease; PR, partial response; CR, complete response.

Supplemental Figure 3

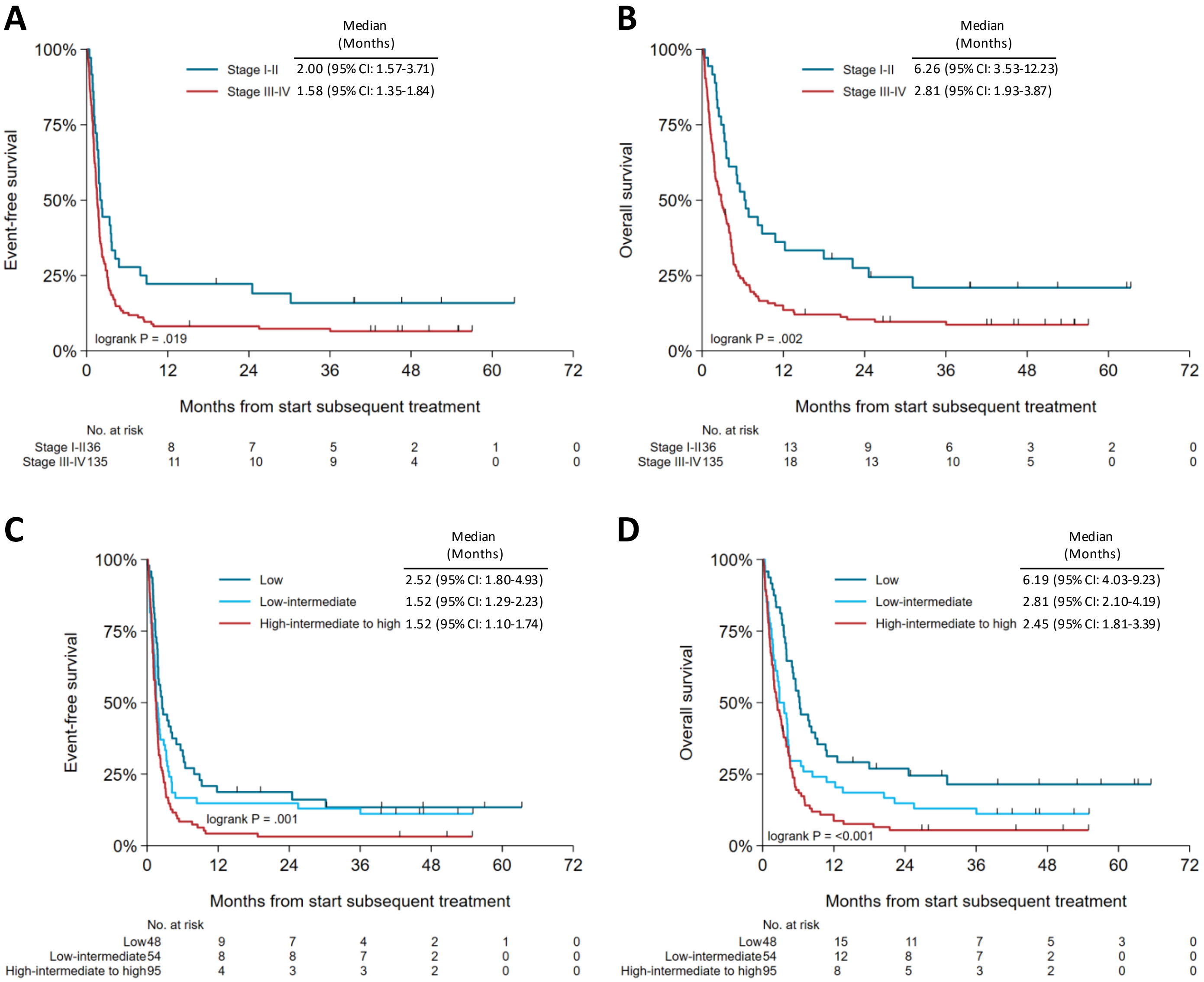

Supplemental Figure 4

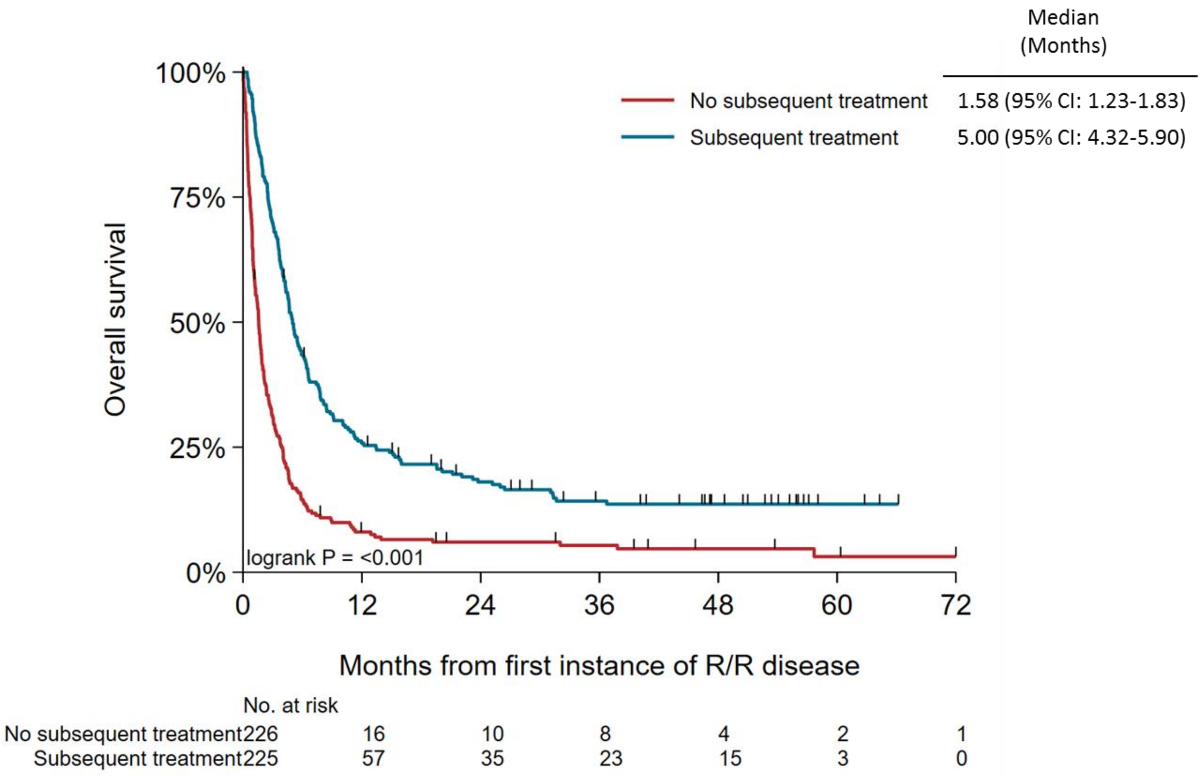

Supplemental Table 1

Supplemental Table 1. Patient characteristics of the patients with long-term survival (alive ≥3 years from start subsequent treatment)

Characteristics	NCR cohort	
	N	(%)
**Total no. of patients**	17	(9)
**Sex, male**	10	(59)
**Age, years**		
Median (min-max)	57 (28-78)	
18-60	7	(41)
61-69	9	(53)
≥70	1	(6)
**Primary diagnosis**		
DLBCL	16	(94)
tFL	0	(0)
PMBCL	1	(6)
Indeterminate/ missing	0	(0)
**Disease stage**		
I-II	6	(35)
III-IV	10	(59)
Unknown	1	(6)
**ECOG performance status**		
0-1	7	(41)
2-4	1	(6)
Unknown	9	(53)
**IPI risk classification**[a]		
Low	7	(41)
Low-intermediate	7	(41)
High-intermediate to high	3	(18)
Missing or incompletely assessed[b]	8	(47)
**R/R disease category**		
Primary refractory	14	(82)
Refractory to ≥ second-line treatment	1	(6)
Relapsed ≤12 months post-autoSCT	2	(12)
**Prior systemic therapy lines**[c]		
1	14	(82)
2-3	1	(6)
≥4	0	(0)
**Center of treatment**		
Non-academic	8	(47)
Academic	9	(53)

Abbreviations: DLBCL, diffuse large B-cell lymphoma; tFL, transformed follicular lymphoma; PMBCL, primary mediastinal B-cell lymphoma. IPI, International Prognostic Index; R/R, relapsed or refractory; autoSCT, autologous stem cell transplantation.

[a]In the advent of a missing IPI risk parameter, that parameter was regarded as negative (i.e. no point assigned) in the NCR dataset. Therefore, an IPI score could be calculated for all patients.

[b]Percentage of patients in whom one or more IPI risk parameters were missing or unknown.

[c]Similar to SCHOLAR-1, information on prior systemic therapy lines is only presented for the 88% (n=15) of patients who were refractory to systemic therapy and not for the patients who relapsed post-autoSCT (n=2). Percentages in the Table are shown as percentage of the total cohort (N=17). Of the 15 patients who were refractory to systemic treatment, 93% (n=14) received 1 prior systemic therapy line and 7% (n=1) received 2-3 prior systemic therapy lines.

Supplemental Table 2

Supplemental Table 2. Univariable and multivariable analyses for event-free survival (EFS) and overall survival (OS)

	Univariable						Multivariable			
	EFS			OS			EFS		OS	
	**Events/N**	**HR (95% CI)**	***P*-value**	**Events/N**	**HR (95% CI)**	***P*-value**	**HR (95% CI)**	***P*-value**	**HR (95% CI)**	***P*-value**
**Sex**										
Male	116/125	1.00		112/125	1.00		1.00		-	
Female	65/72	0.73 (0.54-0.99)	0.047	62/72	0.82 (0.60-1.12)	0.218	0.78 (0.55-1.10)	0.156	-	-
**Age, years**										
<60	74/81	1.00		70/81	1.00		-		-	
≥ 60	107/116	1.05 (0.78-1.41)	0.769	104/116	1.16 (0.86-1.57)	0.338	-		-	-
**Primary diagnosis**										
DLBCL	155/171	1.00		149/171	1.00		-		-	
tFL	19/19	1.14 (0.71-1.85)	0.585	19/19	1.35 (0.83-2.20)	0.219	-		-	-
PMBCL	7/7	1.26 (0.59-2.70)	0.547	6/7	0.89 (0.39-2.03)	0.796	-		-	-
**Disease stage**										
I-II	30/36	1.00		28/36	1.00		1.00		1.00	
III-IV	126/135	1.61 (1.08-2.40)	0.020	122/135	1.90 (1.25-2.87)	0.002	0.95 (0.55-1.66)	0.861	1.15 (0.66-2.00)	0.631
**IPI risk classification**										
Low	41/48	1.00		37/48	1.00		1.00		1.00	
Low-intermediate	48/54	1.39 (0.91-2.11)	0.123	48/54	1.65 (1.08-2.54)	0.022	1.37 (0.78-2.41)	0.275	1.63 (0.91-2.93)	0.098
High-intermediate to high	92/95	1.96 (1.35-2.85)	<0.001	89/95	2.27 (1.54-3.35)	<0.001	1.98 (1.08-3.61)	0.027	2.24 (1.20-4.18)	0.011
**R/R disease category**										
Primary refractory	85/97	1.00		83/97	1.00		1.00		1.00	
Refractory to ≥ second-line treatment	72/73	1.20 (0.76-1.90)	0.424	68/73	1.20 (0.76-1.91)	0.439	0.85 (0.50-1.43)	0.538	1.11 (0.65-1.90)	0.701
Relapsed ≤12 months post-autoSCT	24/27	1.78 (1.11-2.84)	0.016	23/27	1.61 (0.99-2.59)	0.050	1.22 (0.71-2.10)	0.468	1.38 (0.80-2.39)	0.243

Abbreviations: DLBCL, diffuse large B-cell lymphoma; tFL, transformed follicular lymphoma; PMBCL, primary mediastinal B-cell lymphoma. IPI, International Prognostic Index; R/R, relapsed or refractory; autoSCT, autologous stem cell transplantation; EFS, event-free survival; OS, overall survival; HR, hazard ratio; 95% CI, 95% confidence interval.

Supplemental Table 3

Supplemental Table 3. Patient characteristics of the 455 patients selected from the NCR meeting relapsed or refractory (R/R) disease criteria presented for patients with or without initiation of subsequent treatment at the first instance of R/R disease

**Characteristics**[a]	**Subsequent treatment**		**No subsequent treatment**		***P*-value**[b]
	**N**	**(%)**	**N**	**(%)**	
**Total no. of patients**	225	(100)	230	(100)	
**Sex, male**	144	(64)	133	(58)	0.180
**Age, years**					
Median (min-max)	62 (18-81)		71 (18-89)		<0.001
18-60	105	(47)	48	(21)	
61-69	74	(33)	54	(23)	<0.001
≥70	46	(20)	128	(56)	
**Primary diagnosis**					
DLBCL	199	(88)	203	(88)	
tFL	19	(8)	24	(10)	0.347
PMBCL	7	(3)	3	(1)	
Indeterminate/ missing	0	(0)	0	(0)	
**Disease stage**					
I-II	49	(22)	45	(20)	
III-IV	161	(72)	170	(74)	0.846
Unknown	15	(7)	15	(7)	
**ECOG performance status**					
0-1	112	(50)	72	(31)	
2-4	10	(4)	37	(16)	<0.001
Unknown	103	(46)	121	(53)	
**IPI risk classification**[c]					
Low	58	(26)	40	(17)	
Low-intermediate	54	(24)	56	(24)	<0.001
High-intermediate to high	113	(50)	134	(58)	
Missing or incompletely assessed[d]	98	(44)	99	(43)	
**R/R disease category**					
Primary refractory	107	(48)	99	(43)	
Refractory to ≥ second-line treatment	83	(37)	116	(50)	0.001
Relapsed ≤12 months post-autoSCT	35	(16)	15	(7)	
**Prior systemic therapy lines**[e]					
1	107	(48)	99	(43)	
2-3	82	(36)	114	(50)	0.169
≥4	1	(<1)	2	(1)	
**Center of treatment**					
Non-academic	179	(80)	203	(88)	0.015
Academic	46	(20)	27	(12)	

Abbreviations: DLBCL, diffuse large B-cell lymphoma; tFL, transformed follicular ymphoma; PMBCL, primary mediastinal B-cell lymphoma. IPI, International Prognostic Index; R/R, relapsed or refractory; autoSCT, autologous stem cell transplantation.
[a]Baseline characteristics were analyzed for the therapy line corresponding with the first instance of R/R disease (e.g., Baseline characteristics for patients with primary refractory disease were analyzed at initiation of first-line treatment).
[b]The *P*-value was calculated with the Fisher's exact test for categorical variables and the Mann-Whitney U test for continuous variables.
[c]In the advent of a missing IPI risk parameter, that parameter was regarded as negative (i.e. no point assigned) in the NCR dataset. Therefore, an IPI score could be calculated for all patients.
[d]Percentage of patients in whom one or more IPI risk parameters were missing or unknown.
[e]Similar to SCHOLAR-1, information on prior systemic therapy lines is only presented for the 84% (n=190) and 93% (n=215) of patients who were refractory to systemic therapy in the subsequent and no subsequent treatment cohort, respectively, and not for the patients who relapsed post-autoSCT (n=35 and n=15, respectively). Percentages in the Table are shown as percentage of the total cohort (N=225 and N=230, respectively). Of the 190 patients who were refractory to systemic treatment in the subsequent treatment cohort, 56% (n=107) received 1 prior systemic therapy line, 43% (n=82) received 2-3 prior systemic therapy lines and 1% (n=1) received ≥4 prior systemic therapy lines. Of the 215 patients who were refractory to systemic treatment in the subsequent treatment cohort, 46% (n=99) received 1 prior systemic therapy line, 53% (n=114) received 2-3 prior systemic therapy lines and 1% (n=2) received ≥4 prior systemic therapy lines.

Supplemental Table 4

Supplemental Table 4. Patient characteristics according to center of treatment

Characteristics	NCR cohort		Non-academic treatment center		Academic treatment center		*P*-value[a]
	N	(%)	N	(%)	N	(%)	
**Total no. of patients**	197	(100)	131	(100)	66	(100)	
**Sex, male**	125	(63)	79	(60)	46	(70)	0.213
**Age, years**							
Median (min-max)	62 (18-81)		65 (18-81)		56.5 (25-81)		0.003
18-60	87	(44)	48	(37)	39	(59)	
61-69	64	(32)	46	(35)	18	(27)	0.007
≥70	46	(23)	37	(28)	9	(14)	
**Primary diagnosis**							
DLBCL	171	(87)	114	(87)	57	(86)	
tFL	19	(10)	11	(8)	8	(12)	0.462
PMBCL	7	(4)	6	(5)	1	(2)	
Indeterminate/ missing	0	(0)	0	(0)	0	(0)	
**Disease stage**							
I-II	36	(18)	24	(18)	12	(18)	
III-IV	135	(69)	93	(71)	42	(64)	0.346
Unknown	26	(13)	14	(11)	12	(18)	
**ECOG performance status**							
0-1	55	(28)	30	(23)	25	(38)	
2-4	21	(11)	13	(10)	8	(12)	0.050
Unknown	121	(61)	88	(67)	33	(50)	
**IPI risk classification[b]**							
Low	48	(24)	26	(20)	22	(33)	
Low-intermediate	54	(27)	43	(33)	11	(17)	0.052
High-intermediate to high	95	(48)	62	(47)	33	(50)	
Missing or incompletely assessed[c]	119	(60)	84	(64)	35	(53)	
**R/R disease category**							
Primary refractory	97	(49)	68	(52)	29	(44)	
Refractory to ≥ second-line treatment	73	(37)	46	(35)	27	(41)	0.567
Relapsed ≤12 months post-autoSCT	27	(14)	17	(13)	10	(15)	
**Prior systemic therapy lines[d]**							
1	97	(49)	68	(52)	29	(44)	
2-3	73	(37)	46	(35)	27	(41)	0.549
≥4	0	(0)	0	(0)	0	(0)	
**Center of treatment**							
Non-academic	131	(66)	131	(100)	-		-
Academic	66	(34)	-		66	(100)	

Abbreviations: DLBCL, diffuse large B-cell lymphoma; tFL, transformed follicular ymphoma; PMBCL, primary mediastinal B-cell lymphoma. IPI, International Prognostic Index; R/R, relapsed or refractory; autoSCT, autologous stem cell transplantation.

[a]The *P*-value was calculated with the Fisher's exact test for categorical variables and the Mann-Whitney U test for continuous variables.

[b]In the advent of a missing IPI risk parameter, that parameter was regarded as negative (i.e. no point assigned) in the NCR dataset. Therefore, an IPI score could be calculated for all patients.

[c]Percentage of patients in whom one or more IPI risk parameters were missing or unknown.

[d]Similar to SCHOLAR-1, information on prior systemic therapy lines is only presented for the 86% (n=170), 87% (n=114) and 85% (n=56) of patients who were refractory to systemic therapy in the NCR, non-academic treatment center and academic treatment center cohort, respectively, and not for the patients who relapsed post-autoSCT (n=27, n=17 and n=10, respectively). Percentages in the Table are shown as percentage of the total cohorts (N=197, N=131 and N=66, respectively). Of the 170 patients who were refractory to systemic treatment in the NCR cohort, 57% (n=97) received 1 prior systemic therapy line and 43% (n=73) received 2-3 prior systemic therapy lines. Of the 114 patients who were refractory to systemic treatment in the non-academic treatment center cohort, 60% (n=68) received 1 prior systemic therapy line and 40% (n=46) received 2-3 prior systemic therapy lines. Of the 66 patients who were refractory to systemic treatment in the academic treatment center cohort, 52% (n=29) received 1 prior systemic therapy line and 48% (n=27) received 2-3 prior systemic therapy lines.

Supplemental Table 5

Supplemental Table 5. Patient characteristics per relapsed or refractory (R/R) disease subgroup

Characteristics	NCR cohort		Primary refractory		Refractory to ≥ second-line treatment		Relapsed ≤12 months post-autoSCT		*P*-value[a]
	N	(%)	N	(%)	N	(%)	N	(%)	
**Total no. of patients**	197	(100)	97	(100)	73	(100)	27	(100)	
**Sex, male**	125	(63)	65	(67)	46	(63)	14	(52)	0.335
**Age, years**									
Median (min-max)	62 (18-81)		65 (18-81)		60 (25-81)		62 (32-75)		0.207
18-60	87	(44)	36	(37)	38	(52)	13	(48)	
61-69	64	(32)	38	(39)	15	(21)	11	(41)	0.040
≥70	46	(23)	23	(24)	20	(27)	3	(11)	
**Primary diagnosis**									
DLBCL	171	(87)	87	(90)	57	(78)	27	(100)	
tFL	19	(10)	7	(7)	12	(16)	0	(0)	0.046
PMBCL	7	(4)	3	(3)	4	(5)	0	(0)	
Indeterminate/ missing	0	(0)	0	(0)	0	(0)	0	(0)	
**Disease stage**									
I-II	36	(18)	20	(21)	13	(18)	3	(11)	
III-IV	135	(69)	70	(72)	48	(66)	17	(63)	0.093
Unknown	26	(13)	7	(7)	12	(16)	7	(26)	
**ECOG performance status**									
0-1	55	(28)	26	(27)	19	(26)	10	(37)	
2-4	21	(11)	11	(11)	8	(11)	2	(7)	0.863
Unknown	121	(61)	60	(62)	46	(63)	15	(56)	
**IPI risk classification[b]**									
Low	48	(24)	19	(20)	20	(27)	9	(33)	
Low-intermediate	54	(27)	31	(32)	17	(23)	6	(22)	0.558
High-intermediate to high	95	(48)	47	(48)	36	(49)	12	(44)	
Missing or incompletely assessed[c]	119	(60)	59	(61)	46	(63)	14	(52)	
**R/R disease category**									
Primary refractory	97	(49)	97	(100)	-		-		
Refractory to ≥ second-line treatment	73	(37)	-		73	(100)	-		-
Relapsed ≤12 months post-autoSCT	27	(14)	-		-		27	(100)	
**Prior systemic therapy lines[d]**									
1	97	(49)	97	(100)	0	(0)	-		
2-3	73	(37)	0	(0)	73	(100)	-		-
≥4	0	(0)	0	(0)	0	(0)	-		
**Center of treatment**									
Non-academic	131	(66)	68	(70)	46	(63)	17	(63)	0.567
Academic	66	(34)	29	(30)	27	(37)	10	(37)	

Abbreviations: DLBCL, diffuse large B-cell lymphoma; tFL, transformed follicular lymphoma; PMBCL, primary mediastinal B-cell lymphoma. IPI, International Prognostic Index; R/R, relapsed or refractory; autoSCT, autologous stem cell transplantation.

[a]The *P*-value was calculated with the Fisher's exact test for categorical variables and Kruskal-Wallis test for continuous variables.

[b]In the advent of a missing IPI risk parameter, that parameter was regarded as negative (i.e. no point assigned) in the NCR dataset. Therefore, an IPI score could be calculated for all patients.

[c]Percentage of patients in whom one or more IPI risk parameters were missing or unknown.

[d]Similar to SCHOLAR-1, information on prior systemic therapy lines is only presented for the 86% (n=170) of patients who were refractory to systemic therapy and not for the patients who relapsed post-autoSCT (n=27). Percentages in the Table are shown as percentage of the total cohort (N=197). Of the 170 patients who were refractory to systemic treatment, 57% (n=97) received 1 prior systemic therapy line and 43% (n=73) received 2-3 prior systemic therapy lines.

Supplemental Figure legends

Supplemental Figure 1. Patient inclusion from the NCR. The presented flowchart visualizes the patient inclusion from the NCR using criteria similar to SCHOLAR-1. Patients were included at the first instance of relapsed or refractory disease.

Supplemental Figure 2. Best response to subsequent treatment per relapsed or refractory (R/R) disease subgroup. The best response to subsequent treatment at the first instance of R/R disease according to the R/R disease subgroup and for the total cohort is shown.

Supplemental Figure 3. Event-free survival (EFS) and overall survival (OS) according to disease stage (A and B, respectively) and International Prognostic Index (IPI) score (C and D, respectively). EFS and OS from start of subsequent treatment by disease stage (stage I-II vs. III-IV) and IPI score (Low vs. Low-intermediate vs. High-intermediate to high) are presented.

Supplemental Figure 4. Overall survival (OS) from the first instance of relapsed or refractory (R/R) disease for the total* cohort selected from the NCR meeting R/R disease criteria. The blue line indicates the OS of patients who did receive subsequent treatment and the red line the OS of patients who did not receive subsequent treatment.

*For 4 patients in the no subsequent treatment group overall survival could not be estimated as the date of first instance of R/R disease was unknown, therefore these patients were excluded from the survival analysis.
